# Post-resonance backward whirl analysis in cracked overhung rotors

**DOI:** 10.1038/s41598-022-12068-w

**Published:** 2022-05-20

**Authors:** Tariq Alzarooni, Mohammad AL-Shudeifat, Oleg Shiryayev, C. Nataraj

**Affiliations:** 1 Department of Mechanical Engineering, Khalifa University of Science and Technology, Main Campus, PO Box 127788, Abu Dhabi, United Arab Emirates; 2 Department of Aerospace Engineering, Khalifa University of Science and Technology, Main Campus, PO Box 127788, Abu Dhabi, United Arab Emirates; 3 Department of Mechanical Engineering, University of Alaska Anchorage, 3211 Providence Dr., Anchorage, AK 99508 USA; 4 Villanova Center for Analytics of Dynamic Systems, Villanova University, 800 Lancaster Ave., Villanova, PA 19085 USA

**Keywords:** Aerospace engineering, Mechanical engineering

## Abstract

Overhung rotors usually exhibit recurrent transitions through critical whirl rotational speeds during startup and coast down operations, which significantly differ from their steady-state whirl responses. The presence of angular acceleration results in a linear-time-varying (LTV) system, which, although technically linear, still presents complexities often evinced by a nonlinear system. In general, backward whirl zones can either precede the critical forward whirl speed (termed as pre-resonance backward whirl, Pr-BW), or immediately follow the critical forward whirl speed (termed as post-resonance backward whirl, Po-BW). The Po-BW in the whirl response of a cracked overhung rotor with a breathing crack is studied here as distinct from that of geometrically symmetric configurations of other rotor systems. The equations of motion from the finite element (FE) model of an overhung rotor system with a breathing crack are numerically integrated to obtain the whirl response. The transient whirl responses with different bearing conditions are thoroughly investigated for excitation of Po-BW. The Po-BW zones of rotational speeds are determined via the wavelet transform method and full spectrum analysis (FSA) and applied to signals with added noise. The results of this work confirm the excitation of the Po-BW in cracked overhung rotors and confirm the robustness of the employed methods.

## Introduction

Rotordynamic systems are employed in several heavy-duty aerospace and industrial systems such as aircraft engines, helicopter shafts, electric power generation units, turbines, compressors, and pumps. Such mission-critical applications of rotating systems dictate, as the first priority, the development of early fault detection methodologies to minimize or evade catastrophic failures, some of which could be life threatening and cause a major economic burden. Propagation of transverse cracks in such rotor systems is one of the major causes of damages in a rotordynamic systems. Accordingly, studying the dynamic behavior of cracked rotor systems supported by isotropic and anisotropic bearings has gained broad interest amongst many researchers since 1960’s.

In the literature, two different crack models are employed to analyze the dynamic response of a cracked shaft, which are referred to as fatigue and open crack models^1^. The open crack model is a classical approach, which represents a crack with a fixed depth and position over the entire time span of a rotor’s rotation cycle. The fatigue crack model can be used to represent alternating crack configurations throughout the rotor revolution due to rotor inertia and/or unbalance excitation forces. The fatigue crack model has further been subcategorized into switching and breathing cracks based on the crack opening/closing mechanism within a complete shaft revolution. The switching, or hinged crack model, was first introduced by Gasch^2^ and it simulates fatigue crack behavior via abrupt stiffness change from a fully-open to a fully-closed crack configuration at certain shaft rotation angles during a full revolution cycle. On the other hand, a breathing crack model proposed by Mayes and Davies^3^ simulates the behavior of a fatigue crack via gradual opening and closure over a revolution cycle. Considering intensive unbalance excitation and static loads of the rotor system, the breathing crack model replicates real world rotor behavior more accurately. Consequently, the breathing crack model has been chosen for the present work due to the above rationale.

Breathing crack models have been extensively investigated by many researchers since 1980’s^4–38^. Vast majority of research work have expressed the behavior of the breathing crack mathematically in the form of stiffness variation using a simple cosine function^4–9^, originally proposed by Mayes and Davies^3^. In the work of Cheng et al.^7^, a strain energy release rate method based on the Mayes and Davies cosine function was used to develop the breathing crack model without assuming weight (inertial) dominance mechanism for a Jeffcott rotor. The crack opening and closure state was determined based on the angle between the crack direction and the shaft deformation. Accordingly, it was reported that the nonlinear rotor can exhibit lower whirling amplitudes compared to the rotor with an open crack or even the intact rotor. Other researchers have expressed the breathing crack mechanism in the form of a truncated cosine function to simulate time-varying flexibility^10–14,38–40^.

Al-Shudeifat et al.^15,17^ have developed three new breathing functions, which were based on the Fourier series expansion to represent the time varying moment of inertia for the cracked element. This, in turn, is used in formulating the actual time-varying stiffness matrix of the cracked element. These functions were derived to approximate expressions for area moments of inertia about horizontal and vertical centroidal axes. The same breathing functions were also used by other researchers to analyze the response behavior of cracked rotor systems^16,34,36^. Linear Fracture Mechanics (LFM) theories have also been widely employed for modeling a breathing crack in the shaft^13,18–26,37,38^. In this approach, the flexibility matrix is first derived using Castigliano’s theorem. Then, additional flexibility is superimposed on the cracked section using supplementary compliances. It follows that breathing cracks are simulated based on varying stress intensity factors (SIF) along the crack edge using a coordinate transformation matrix for bending moment parameters via integration methods. SIF’s sign convention was used to imply the crack’s state: positive SIF implied crack’s openness, whereas negative SIF implied crack’s closure^13,18–25^. Further, a few researchers have used truncated cosine series to obtain linear-time periodic varying stiffness of strain energy rate-based crack model^38^. These fracture mechanics formulations along with linear varying moments of inertia are considered to be superior compared to classical hinged or breathing cosine-based function models because stiffness variation can also be correlated to the depth of crack.

Whirl orbit analysis have also been studied in depth for determining potential vibration characteristics that signify the presence of the breathing crack phenomena^7,17,21,24^. Dynamic whirl response studies have been performed by Darpe et al.^24^ via analytical and experimental analyses of orbital plots of a cracked rotor during passage through 1/2, 1/3, and 1/5 critical speeds, using a transient operation model. In addition, important contrast has been drawn in the paper regarding different influences of breathing crack, switching crack, open crack and crack-free models on peak resonance amplitude and whirling analysis. However, there were no studies performed on the whirl reversal phenomena. Al-Shudeifat and Butcher^17^ studied the orbit loops against pre-resonance BW and FW frequencies. A Finite Element model with harmonic balance (HB) solution was used to obtain the orbital response of the rotor. Pairs of subcritical peak responses were observed at 1/2, 1/3, and 1/4 of the first critical speed. FW was seen to precede BW at each subcritical response. Further, the shift in subcritical forward and backward whirl speeds to the lower side was also observed as the crack depth increased. It was also reported that the angle of orientation of the unbalance force with respect to the transverse crack and the number of associated loops can be decisively used for rotor crack diagnosis. Experimental analysis was also employed to verify numerical findings of whirl analysis. Cheng et al.^7^ developed a cracked Jeffcott rotor model and analyzed the whirl response at the critical speed. It was reported that the whirl response of a rotor is independent of the unbalance orientation angle.

More details regarding sequential changes of shaft whirl with a breathing crack in the vicinity of the critical speed range was discussed by Jun and Gadala^21^. A fourth-order partial differential equation of elastodynamic behavior of a thick uniform shaft was developed by considering the rotatory inertia and shear deformation of the cross-section. The gyroscopic effect due to rotation was also considered. The paper concluded that in a normal case the whirl direction coincides with the shaft spin direction (FW). It was reported that an intermittent whirl reversal to BW precession takes place near the critical speed range, which was attributed to the presence of crack. However, it was stated that such phenomena cannot be experimentally captured due to unbalance excitation forces, which preclude whirl reversal observed in the simulation.

Gunter^41^ used FE analysis to compute complex eigenvalues of an overhung rotor with asymmetrical bearing supports. It was shown that bearing asymmetry has resulted in a substantial excitation of the second pre-resonance BW critical speed in addition to first pre-resonance BW critical speed due to unbalance forces. On the contrary, the second pre-resonance critical BW was not reported to be seen when bearing symmetry is maintained. A simultaneous pre-resonance BW and FW precession have been studied by Muszynska^42^ considering an overhung unbalanced rotor supported by flexible anisotropic bearings. In that work, the unbalance-mass along with the shaft bow were simulated at different parts of the rotor, which led to the simultaneous pre-resonance BW and FW precession of different sections of the rotor. This phenomenon was reported to be sensitive to damping, bearing anisotropy, forcing functions magnitudes, and most significantly, their angular orientations. Increasing the damping values was reported to alleviate such phenomena.

The effect of gravity and friction on an overhung disk with rotor–stator contact were explored using the 2-DOF lumping method^43,44^. Dynamic response was obtained using numerical simulation. Rotor stiffening was observed, which lead to a rubbing effect that induced a shift in pre-resonance BW and FW frequencies^43^. Further, it was reported that for a highly stiffened system (due to shaft/stator rub or friction), the effect of gravity can be neglected. In the following publication^44^ it was reported that the extent of pre-resonance BW zones expands with increased eccentricity and coefficient of friction. The study also showed that the frequency content of pre-resonance BW solutions shifts toward higher values due to increased stiffness due to friction contact.

Transient analysis of rotor dynamic systems is essential to capture information related to instantaneous behavior of the rotor, especially in the vicinity of the critical speed zone. This information can be presented in the form of unique dynamic characteristics that are attributed to the presence of transverse breathing cracks in the rotor. In several studies^8,9,17^ the HB method was employed to evaluate changes in the shape of the orbit during passages at 1/3 or 1/2 of resonance speed for transient rotor operations. It was reported that these findings can be employed as important characteristics for detecting the presence of transverse cracks. In other studies^24,37,39,45–48^, numerical solution was employed to solve linear-time-variant (LTV) equations of motion (EOM). A constant acceleration was assumed to represent the start-up operation, which would result in a linear-time variant stiffness. Several studies were performed using FE models^45,46^, whereas a 4-DOF model was used by Fu et al.^48^ as an enhanced feature to a simple 2-DOF Jeffcott model incorporating the gyroscopic effects. In the work of Fu et al.^48^, transient rotor behavior was studied along with incorporation of random and interval uncertainties as an additional feature. Numerical integration of time-variant EOM showed that the transient responses of the rotor are affected by the induced uncertainties. A few other researchers explored various algorithms to optimize the cost of calculation with minimal impact on accuracy. In the study by Subbiah and Rieger^49^, differences as high as 6% were reported if compared to solutions obtained by employing the Runge–Kutta method. Another research group employed Houbolt algorithm to compute the time-varying whirl response of a rotor represented by an FE model with an embedded breathing crack while the rotor accelerated through the critical speed zone^39,47,50^. The breathing crack was represented by a time-varying stiffness matrix expressed in terms of a truncated cosine series. Results indicated that the presence of the crack affects the overall vibration amplitude during the passage through the critical speed. Houbolt algorithm was also employed in another study to examine the dynamic response of a cracked shaft as it undergoes transient start-up operation^51^. A switching (hinged) crack model was used, and it was reported that the dynamic response is affected by crack depth, rotor angular acceleration, and unbalance orientation with respect to the transverse direction of the crack.

In the present work, the state-of-art is advanced by analyzing BW and FW precession for an overhung rotor-bearing-disk system undergoing transient response. A fatigue-based breathing crack model developed earlier by AL-Shideifat^15,17^ is employed in the analysis to achieve accurate verisimilitude with the crack’s behavior. FE model is used to develop LTV EOM for accelerating overhung cracked rotor systems, along with a consideration of the different bearings condition scenarios, including isotropy and anisotropy. The model is also implemented for an intact rotor system to map variations of dynamical behavior and existence of Pr-BW and Po-BW phenomena, and to establish a reference platform to distinguish between cracked and crack-free rotors. In addition, the full spectrum analysis (FSA) and wavelet analysis are employed to verify the existence of these BW zones. Finally, the list of symbols and abbreviations is provided in the supplementary materials of the paper

## Overhung rotor system model with a breathing crack

**Figure 1:**
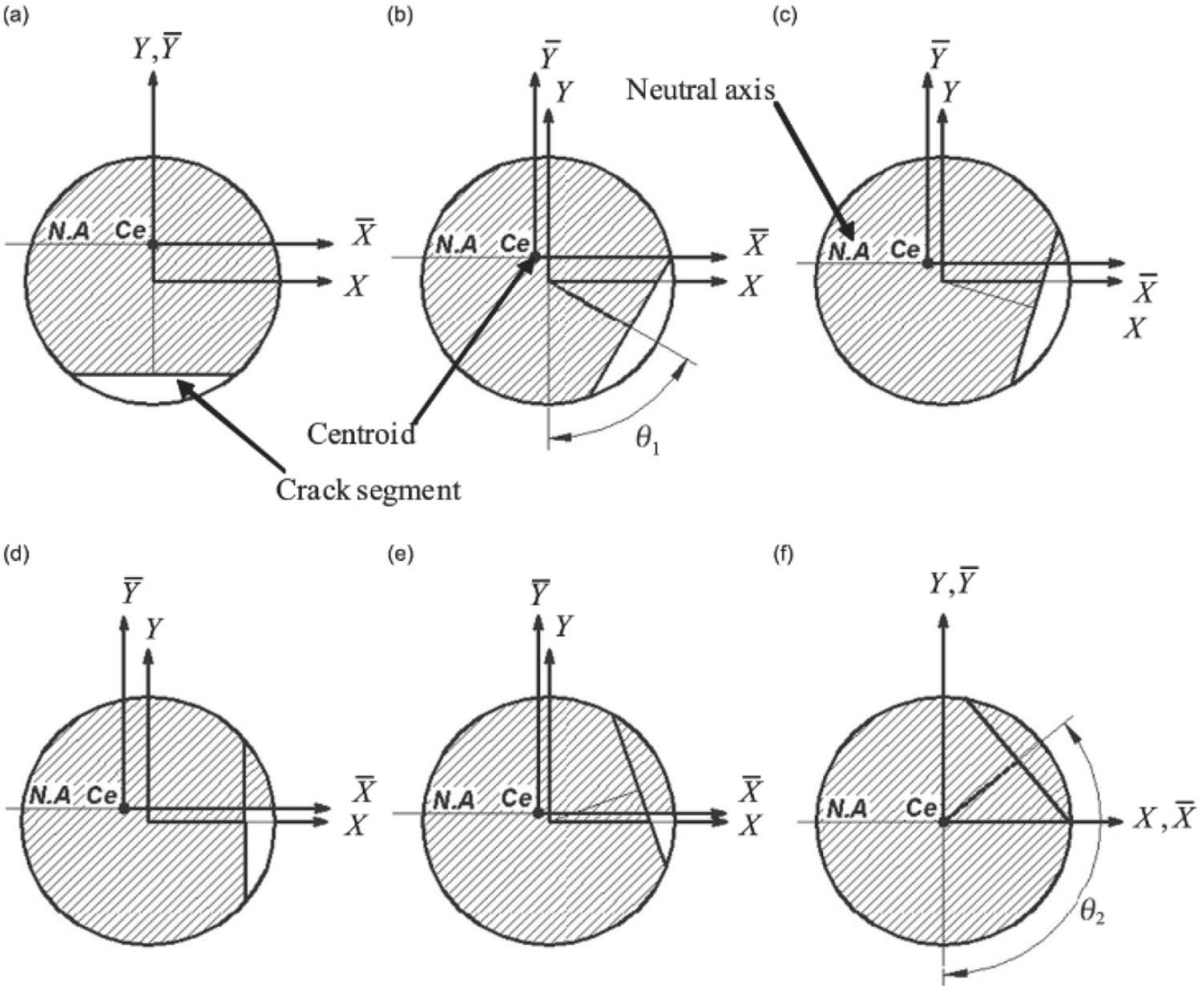
Breathing crack mechanism and relevant shifting centroid^17^.

**Figure 2:**
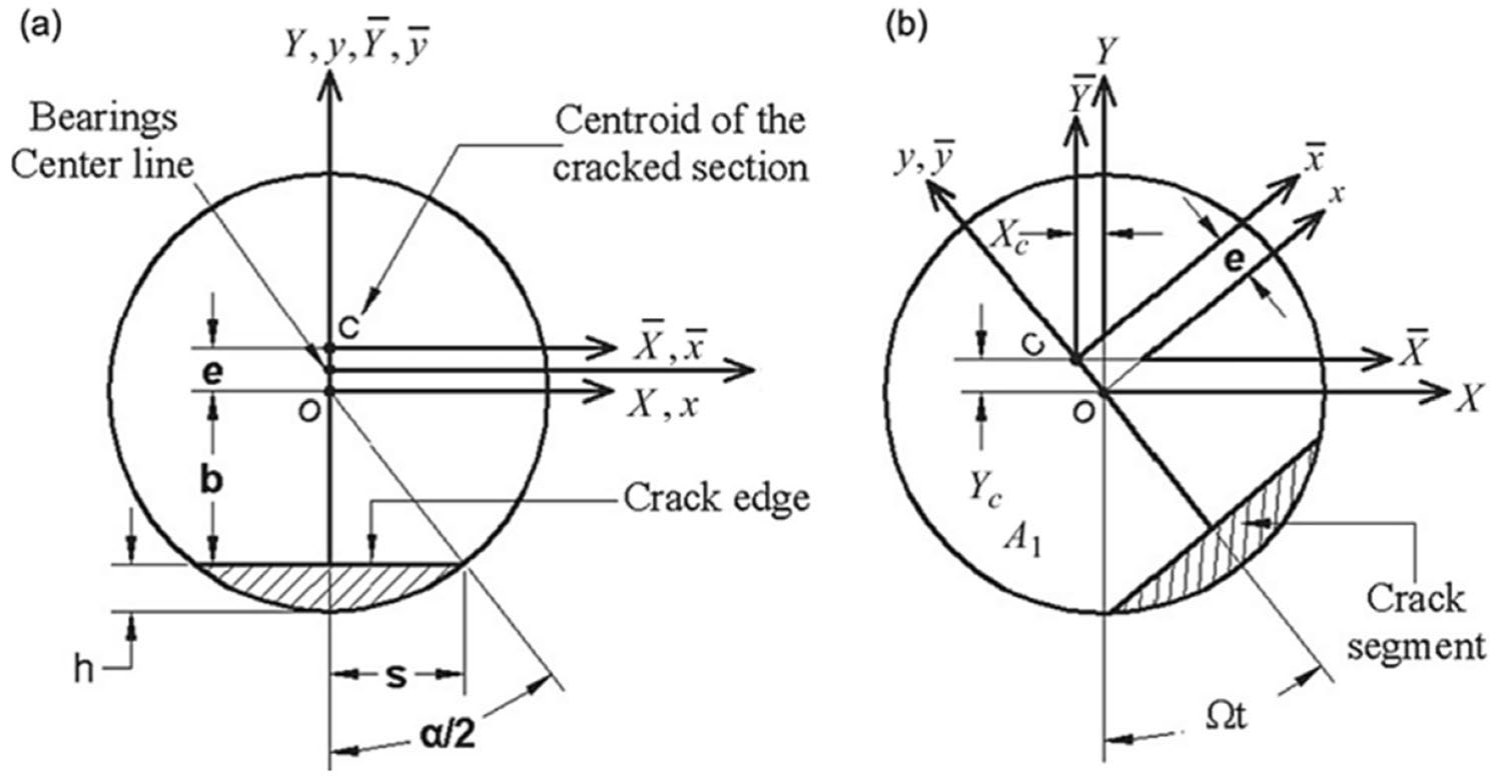
Schematic diagrams of the cracked element cross-section: (**a**) before rotation and (**b**) after the shaft rotates^17^.

The breathing crack model developed earlier^15,17^ is considered here, whose schematic representation is shown in Fig. 1. The crack’s opening and closure mechanism is assumed to be synchronous with the shaft rotational speed of the rotary system. This synchronous crack breathing mechanism is attributed to variation in compression and tension stress states at the periphery of the shaft. The schematic of the shaft’s cracked cross-section is shown in Fig. 2, where the transverse crack depth *h* is normalized by the radius of the shaft *R*, which results in a non-dimensional representation of the crack’s depth *μ* = *h/R*.

**Figure 3:**
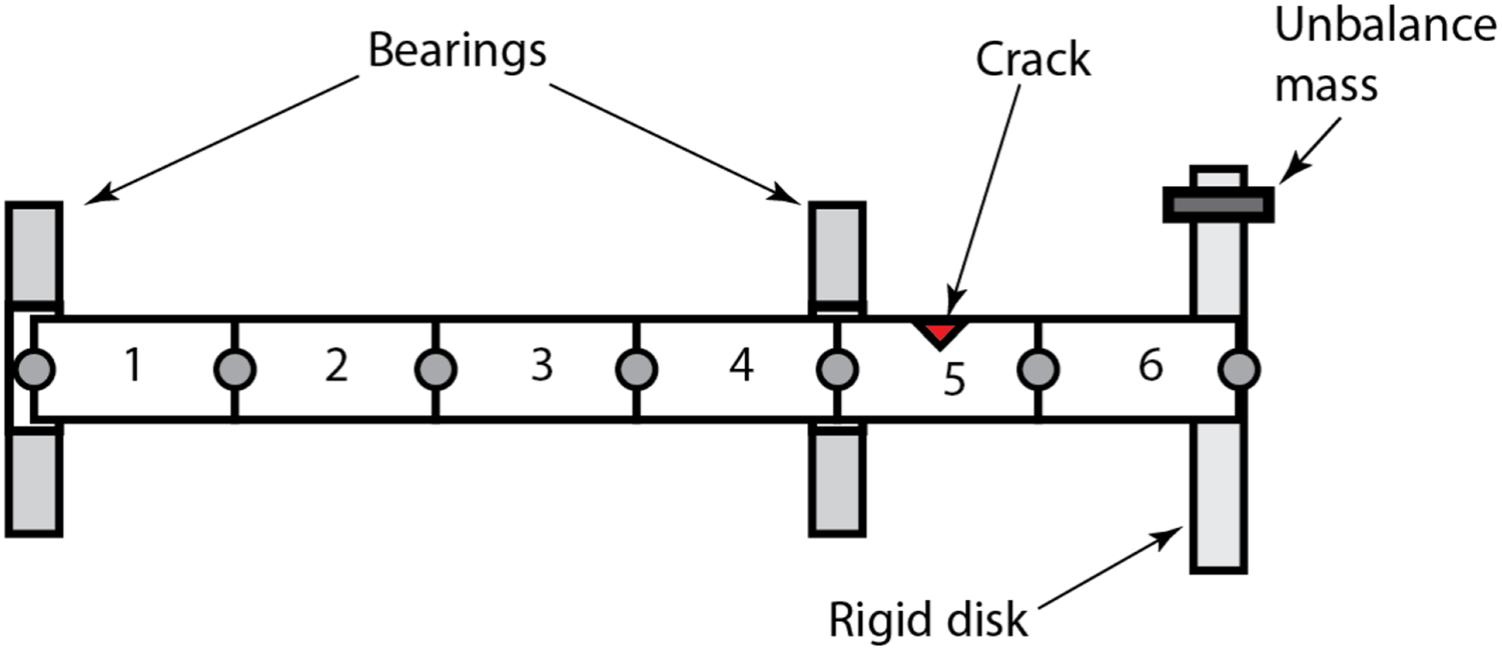
Schematic diagrams of the FE disk-shaft-bearing-rotor system.

The orientation of the unbalance force vector $f_{i}$ is at an angle *β* with respect to the crack opening direction, assuming that the crack opening direction is along the positive X-axis. For a complete revolution cycle starting from the negative Y axis considering the breathing mechanism in Fig. 1, the crack is assumed to be fully open for the range of angles $- \theta_{1} \le \theta \le \theta_{1}$, partially open for $\theta_{1} \le \theta \le \left( {\pi + \alpha } \right)/2$ and $\pi - \alpha /2 \le \theta \le 2\pi - \theta_{1}$ ranges; and, fully closed for $\left( {\pi + \alpha } \right)/2 \le \theta \le \left( {3\pi - \alpha } \right)/2$ . More detailed information on breathing crack functions can be found in the work of AL-Shudeifat and Butcher^17^. The underlying physics of the breathing mechanism is that gradual opening/closure of cracked element leads to variation in location of the centroidal axes of the cracked cross-sectional area, which, in turn, leads to variation in the stiffness of the cracked element. Thus, gradual opening of the crack is modeled by a gradual reduction in shaft stiffness and vice versa. In different terms, this can be described as softening and stiffening effects. Stiffness variation of the cracked element is due to the time-dependent centroidal shift, whereas the remaining intact elements are modeled as Euler–Bernoulli beams with constant circular cross-sections. The schematic shown in Fig. 3 represents the FE model of the considered overhung rotor. The crack is considered to be at the fifth element considering the locations of the maximum shearing force and bending moment on the rotor.

The rotor shaft is treated as an elastic beam, while the disk is modeled as a rigid mass. Two bearings are incorporated using additional horizontal and vertical damping ($c_{xx}$ and $c_{yy}$) and stiffness ($k_{xx}$ and $k_{yy}$) components in the stationary coordinates. These elements, the values of which are provided in Table 1, are merged into the global stiffness and damping matrices of the FE model.

**Table 1:** Physical parameters of the rotor.

Description	Value	Description	Value
Length of the rotor	700 mm	Outer radius of the disk	75 mm
Radius of the rotor	9.5 mm	Inner radius of the disk	9.5 mm
Density of the shaft	7850 kg/m^3^	Thickness of the disk	15 mm
Modulus of elasticity	2.1 × 10^11^ N/m^2^	Mass of the rigid aluminum disk	0.663 kg
Mass unbalance, *me*	5 × 10^11^ kg·m	External damping, *δ*	6 s^−1^
Unbalance force angle, $\beta$	varying	Internal damping, *η*	5 × 10^–7^ s
$k_{xx}$ and $k_{yy}$(isotropic)	10^7^ N/m	$c_{xx}$ and $c_{yy}$	500 N·s/m

The internal material damping of the rotor is assumed to be proportional to the global stiffness matrix ${\mathbf{K}}_{S}$ of the shaft in the rotating coordinates as ${\mathbf{C}}_{R} = \eta {\mathbf{K}}_{S}$, where $\eta$ is the stiffness proportional viscous damping coefficient^38,52,53^. Accordingly, the transformation of this damping matrix into fixed coordinates yields a damping force described as ${\mathbf{F}}_{D} = - \eta \left( {{\mathbf{K}}_{S} {\dot{\mathbf{q}}}\left( t \right) + {\mathbf{K}}_{C} {\mathbf{q}}\left( t \right)} \right)$ where ${\mathbf{K}}_{C}$ is a skew-symmetric circulation matrix^52^. Further, incorporating the angular acceleration rate into the model also adds a skew-symmetric stiffness matrix that is proportional to the gyroscopic matrix as ${\mathbf{K}}_{G} = \alpha {\mathbf{G}}$ where $\alpha$ is the angular acceleration rate of the shaft^54^. Consequently, the equations of motion of the FE model of the considered system are written in matrix form as:

1 $${\mathbf{M}{\ddot{\mathbf{q}}}}\left( t \right) + \left( {\delta {\mathbf{M}} + \eta {\mathbf{K}}_{S} + \Omega (t){\mathbf{G}}} \right){\dot{\mathbf{q}}}\left( t \right) + \left( {{\mathbf{K}}_{T} + \Omega (t)\eta {\mathbf{K}}_{C} + {\mathbf{K}}_{G} } \right){\mathbf{q}}\left( t \right) = {\mathbf{F}}_{u} \left( t \right) + {\mathbf{F}}_{g}$$

where, ${\mathbf{q}}\left( t \right)$ represents the 4(N + 1) vector of translational and rotational displacements of the N + 1 nodes of the FE model, the ${\mathbf{M}}$,${\mathbf{K}}_{S}$,${\mathbf{G}}$ and ${\mathbf{K}}_{T}$ are the global mass, shaft stiffness, gyroscopic and total stiffness (shaft and bearings) matrices, respectively, and $\delta$ is the external mass proportional viscous damping coefficient. The mass matrix ${\mathbf{M}}$ constitutes the sum of rotor’s classical and inertial mass matrices including the rigid disk mass matrices.

The overall system unbalance force vector ${\mathbf{F}}_{u} (t)$ has a size of 4(N + 1) × 1. For the shaft angular displacement and velocity expressed as $\theta \left( t \right) = \alpha t^{2} /2$ and $\Omega \left( t \right) = \alpha t$, respectively, the unbalance force vector will have two components at the $i^{th}$ node, where they are added according to the following relations:

2 $$f_{i} = \left[ {\begin{array}{*{20}c} {f_{i}^{u} } & {f_{i}^{v} } & 0 & 0 \\ \end{array} } \right]$$

3 $$f_{i}^{u} \left( t \right) = me\alpha^{2} t^{2} \cos \left( {\theta \left( t \right) + \beta } \right) - me\alpha \sin \left( {\theta \left( t \right) + \beta } \right)$$

4 $$f_{i}^{v} \left( t \right) = me\alpha^{2} t^{2} \sin \left( {\theta \left( t \right) + \beta } \right) + me\alpha \cos \left( {\theta \left( t \right) + \beta } \right)$$

where *m* is the unbalance mass, *e* is the unbalance mass eccentricity, and *β* is the orientation angle of the unbalance force vector with respect to the transverse crack direction (i.e., it can be the positive X-axis). Therefore, the elements of ${\mathbf{F}}_{u} (t)$ at the node corresponding to the disc location are given in Eqs. (3–4), while the other nodal unbalance forces are set to zero. The gravity force vector is included in ${\mathbf{F}}_{g}$. As noted earlier, bearings were included in the model through the addition of stiffness and damping at the corresponding nodes and incorporating them in the global stiffness and damping matrices.

Analogous to the derivation of the cracked element stiffness matrix in an earlier study^55^, the following elemental stiffness matrix of the cracked element with a breathing crack model is obtained and merged with the $j^{th}$ cracked element matrix of the global stiffness matrix:

5 $${\mathbf{K}}(t) = \frac{E}{{l^{3} }}\left[ {\begin{array}{*{20}l} {12I_{{\overline{Y}}} \left( t \right)} & {12I_{{\overline{X}\overline{Y}}} (t)} & { - 6lI_{{\overline{X}\overline{Y}}} (t)} & {6lI_{{\overline{Y}}} \left( t \right)} & { - 12lI_{{\overline{Y}}} \left( t \right)} & { - 12I_{{\overline{X}\overline{Y}}} (t)} & { - 6lI_{{\overline{X}\overline{Y}}} (t)} & {6lI_{{\overline{Y}}} \left( t \right)} \\ {12I_{{\overline{X}\overline{Y}}} (t)} & {12I_{{\overline{X}}} \left( t \right)} & { - 6lI_{{\overline{X}}} \left( t \right)} & {6lI_{{\overline{X}\overline{Y}}} (t)} & { - 12I_{{\overline{X}\overline{Y}}} (t)} & { - 12I_{{\overline{X}}} \left( t \right)} & { - 6lI_{{\overline{X}}} \left( t \right)} & {6lI_{{\overline{X}\overline{Y}}} (t)} \\ { - 6lI_{{\overline{X}\overline{Y}}} (t)} & { - 6lI_{{\overline{X}}} \left( t \right)} & {4l^{2} I_{{\overline{X}}} \left( t \right)} & { - 4l^{2} I_{{\overline{X}\overline{Y}}} (t)} & {6lI_{{\overline{X}\overline{Y}}} (t)} & {6lI_{{\overline{X}}} \left( t \right)} & {2l^{2} I_{{\overline{X}}} \left( t \right)} & { - 2l^{2} I_{{\overline{X}\overline{Y}}} (t)} \\ {6lI_{{\overline{Y}}} \left( t \right)} & {6lI_{{\overline{X}\overline{Y}}} (t)} & { - 4l^{2} I_{{\overline{X}\overline{Y}}} (t)} & {4l^{2} I_{{\overline{Y}}} \left( t \right)} & { - 6lI_{{\overline{Y}}} \left( t \right)} & { - 6lI_{{\overline{X}\overline{Y}}} (t)} & { - 2l^{2} I_{{\overline{X}\overline{Y}}} (t)} & {2l^{2} I_{{\overline{Y}}} \left( t \right)} \\ { - 12lI_{{\overline{Y}}} \left( t \right)} & { - 12I_{{\overline{X}\overline{Y}}} (t)} & {6lI_{{\overline{X}\overline{Y}}} (t)} & { - 6lI_{{\overline{Y}}} \left( t \right)} & {12I_{{\overline{Y}}} \left( t \right)} & {12I_{{\overline{X}\overline{Y}}} (t)} & {6lI_{{\overline{X}\overline{Y}}} (t)} & { - 6lI_{{\overline{Y}}} \left( t \right)} \\ { - 12I_{{\overline{X}\overline{Y}}} (t)} & { - 12I_{{\overline{X}}} \left( t \right)} & {6lI_{{\overline{X}}} \left( t \right)} & { - 6lI_{{\overline{X}\overline{Y}}} (t)} & {12I_{{\overline{X}\overline{Y}}} (t)} & {12I_{{\overline{X}}} \left( t \right)} & {6lI_{{\overline{X}}} \left( t \right)} & { - 6lI_{{\overline{X}\overline{Y}}} (t)} \\ { - 6lI_{{\overline{X}\overline{Y}}} (t)} & { - 6lI_{{\overline{X}}} \left( t \right)} & {2l^{2} I_{{\overline{X}}} \left( t \right)} & { - 2l^{2} I_{{\overline{X}\overline{Y}}} (t)} & {6lI_{{\overline{X}\overline{Y}}} (t)} & {6lI_{{\overline{X}}} \left( t \right)} & {4l^{2} I_{{\overline{X}}} \left( t \right)} & { - 4l^{2} I_{{\overline{X}\overline{Y}}} (t)} \\ {6lI_{{\overline{Y}}} \left( t \right)} & {6lI_{{\overline{X}\overline{Y}}} (t)} & { - 2l^{2} I_{{\overline{X}\overline{Y}}} (t)} & {2l^{2} I_{{\overline{Y}}} \left( t \right)} & { - 6lI_{{\overline{Y}}} \left( t \right)} & { - 6lI_{{\overline{X}\overline{Y}}} (t)} & { - 4l^{2} I_{{\overline{X}\overline{Y}}} (t)} & {4l^{2} I_{{\overline{Y}}} \left( t \right)} \\ \end{array} } \right]$$

The cross-sectional moments of area $I_{{\overline{X}}}$,$I_{{\overline{Y}}}$ and $I_{{\overline{X}\overline{Y}}}$ are calculated using the breathing functions given in earlier works^15,17^. For details on other matrices in Eq. (1), one is referred to our previous work^55^.

The rotordynamic transient whirl response is obtained by numerical simulation using a constant angular acceleration rate. Consequently, the gyroscopic matrix becomes a time-varying matrix. The resultant whirl amplitude is calculated from $z = \sqrt {u^{2} + v^{2} }$ at the disk node in the FE model where *u* and *v* represent the horizontal and vertical vibration whirl amplitudes, respectively. In addition, both isotropic and anisotropic bearing stiffness conditions are considered in the analysis. The ratio of the vertical stiffness $K_{yy}$ to the horizontal stiffness $K_{xx}$ in the bearings is expressed as $\rho = {{K_{yy} } \mathord{\left/ {\vphantom {{K_{yy} } {K_{xx} }}} \right. \kern-\nulldelimiterspace} {K_{xx} }}$.

## Direction of precession

This method of determining the direction of precession is simply based on computing the vector cross-product of every two consecutive position vectors. The orientation of the resulting vector is used to determine the whirl direction. Accordingly, at consecutive time steps $t_{i}$ and $t_{i + 1}$ the position vectors of the deflection at node $j$ of the FE model are expressed, as ${\mathbf{r}}_{j}^{i} = u_{j}^{i} \hat{i} + v_{j}^{i} \hat{j}$ and ${\mathbf{r}}_{j}^{i + 1} = u_{j}^{i + 1} \hat{i} + v_{j}^{i + 1} \hat{j}$. Therefore, if the direction of the resulting cross-product vector ${\mathbf{r}}_{j}^{i} \times {\mathbf{r}}_{j}^{i + 1}$ is along the positive *z-*axis, then the shaft undergoes FW precession. Otherwise, the shaft undergoes BW precession. Due to its simplicity the method can be reliably applied directly to the whirl response data. However, for more accurate results, the whirl response needs to be centralized to the mean value of the data before the application of the VCP.

## Full spectrum analysis (FSA)

FSA can be used to extract FW and BW frequencies from the shaft’s orthogonal displacements: *u* and *v*. These values are obtained numerically as highlighted in the previous section. However, experimentally based lateral displacements measured via proximity probes can also be used in FSA for the same purpose. According to Muszynska and Goldman^56^, the whirl orbit is represented by a summation of forward and backward rotating vectors $R_{\omega + } e^{{j(\omega t + \overline{\alpha })}} + R_{\omega - } e^{{ - j(\omega t + \overline{\beta })}}$. In this context $R_{\omega + }$ and $R_{\omega - }$ are FW and BW amplitudes, respectively, $\omega$ is the frequency, while $\overline{\alpha }$ and $\overline{\beta }$ are the equivalent phase angles. In brief, FW and BW vector amplitudes are calculated based on the obtained equivalent spectrum lines: $X_{n} e^{{j\alpha_{n} }}$,$X_{n} e^{{ - j\alpha_{n} }}$, $Y_{n} e^{{j\beta_{n} }}$, $Y_{n} e^{{ - j\beta_{n} }}$ using the FFT process. The amplitudes of FW and BW are then calculated as follows:

6 $$\begin{gathered} R_{{\omega_{n} + }} = \sqrt {X_{n}^{2} + Y_{n}^{2} + 2X_{n} Y_{n} \sin (\alpha_{n} - \beta_{n} )} \hfill \\ R_{{\omega_{n} - }} = \sqrt {X_{n}^{2} + Y_{n}^{2} - 2X_{n} Y_{n} \sin (\alpha_{n} - \beta_{n} )} \hfill \\ \end{gathered}$$

## Simulation results and discussion

**Figure 4:**
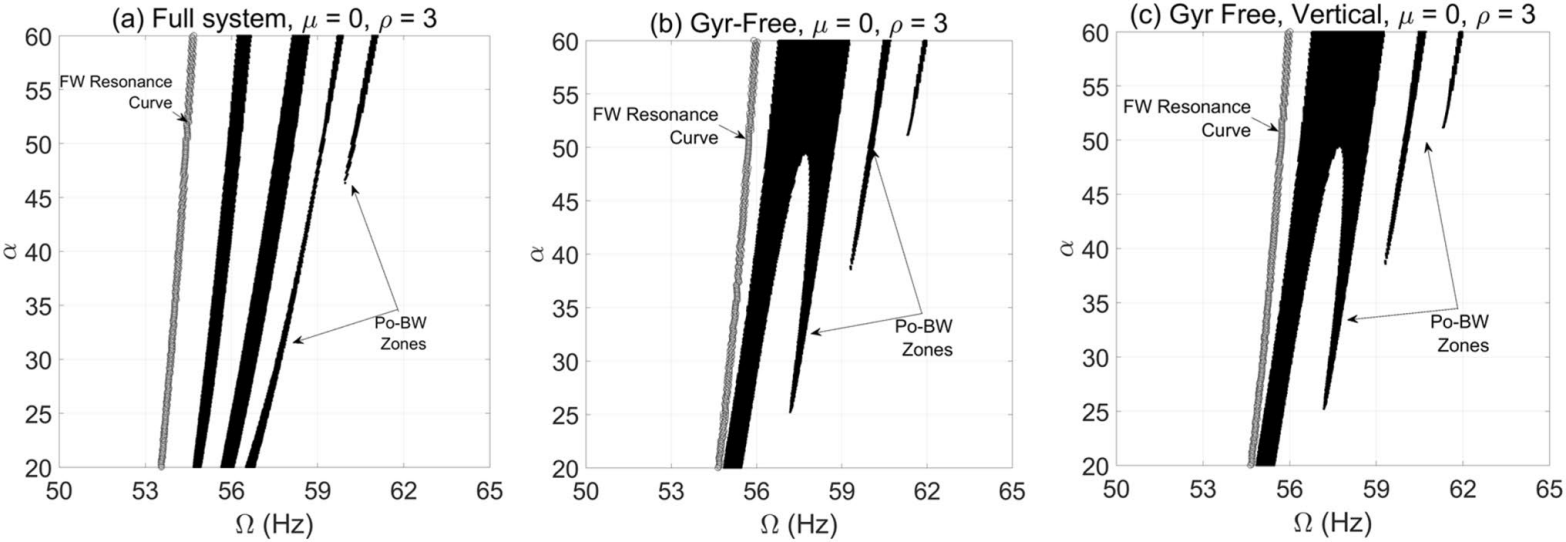
Po-BW zones of rotational speeds at varying angular acceleration rate of overhung intact rotor system with anisotropic bearings including gravity and gyroscopic effects in (**a**); gyroscopic-free system including gravity effect in (**b**); and gravity-free and gyroscopic-free system in (**c**).

The impact of bearing anisotropy and rotor gyroscopic effect on capturing and localizing Pr-BW precession in the rotor-bearing-disk system is well demonstrated in the literature particularly for steady-state operation. For an accelerated rotor system, a new post-resonance backward whirl (Po-BW) was first reported in the work of AL-Shudeifat^45^ by employing the single and double disk rotor system configurations with an open-crack model. These Pr-BW and Po-BW precessions are being evaluated in this context of an accelerating overhung rotor system with a breathing crack. Initially, multiple factors are being considered, including rotor’s gyroscopic effect, bearing’s anisotropy, and gravity to evaluate their impact on Po-BW precessions over a wide-range of angular acceleration rates using the crack-free overhung rotor model. The results are shown in Fig. 4 where VCP was employed to capture these Po-BW zones by iteratively running the overhung rotor simulation at various angular acceleration rates. The full system of Fig. 4a incorporates gyroscopic and gravity factors in the transient model whereas the gyroscopic effect has not been incorporated for Fig. 4b,c. Further, the gravitational factor has been eliminated from the system whose response is shown in Fig. 4c. By comparing the three plots, it can be clearly observed that gyroscopic effect has a major impact on Po-BW excitations. At the same time, it is interesting to note that there are hardly any effects of eliminating the gravitational force when comparing Fig. 4b,c. In addition, it is also observed that Po-BW recurrence and intensities, including their excitation frequencies, vary substantially with angular acceleration rates. Therefore, to conclude from the analysis for transient simulation of intact overhung rotor, it can be stated that the Po-BW zones are mainly affected by the bearing anisotropy, rotor gyroscopic effect, and the angular acceleration rate. It is important to note that gravity will have nearly zero impact on Po-BW excitation zones. One final observation on the crack-free overhung rotor in Fig. 4, the appearance of Pr-BW was not captured over the spanned excitation frequency zone regardless of the gyroscopic effect and gravity.

**Figure 5:**
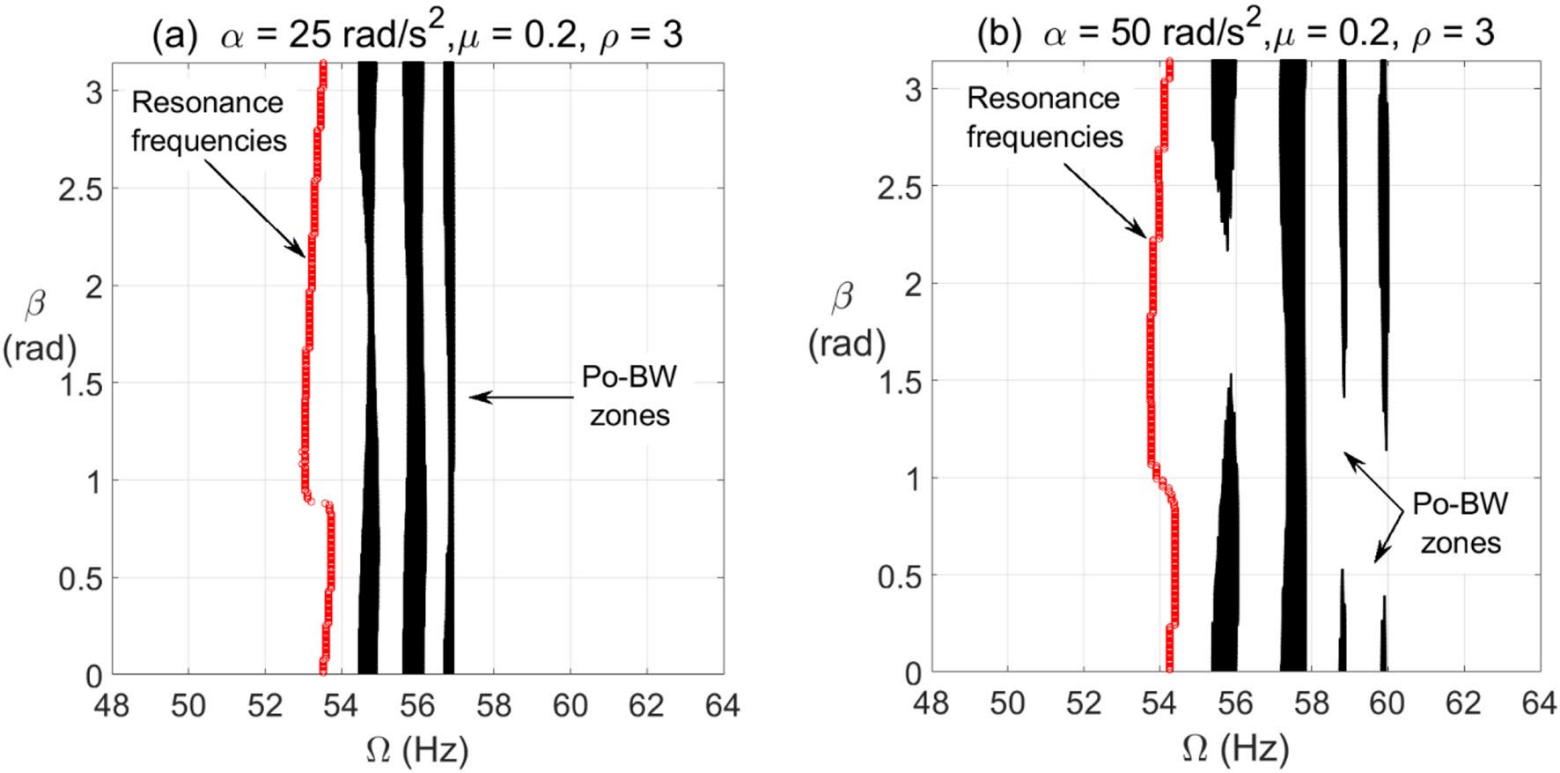
Po-BW zones of rotational speeds at varying unbalance force vector angle of overhung cracked rotor system in (**a**) at $\alpha = 25{\text{ rad/s}}^{2}$ and in (**b**) at $\alpha = 50{\text{ rad/s}}^{2}$ for $\mu = 0.2$ and $\rho = 3$.

For the cracked overhung rotor with a breathing crack model, the influence of unbalance force excitation angle on Po-BW zones have been investigated at different angular acceleration rates. The results are shown in Fig. 5, where the cracked overhung rotor that is equipped with anisotropic bearings at $\rho = 3$ is being evaluated at $\alpha = 25{\text{ rad/s}}^{2}$ and $\alpha = 50{\text{ rad/s}}^{2}$. For the purpose of completeness, it is worth mentioning that gyroscopic and gravity effects have been considered while simulating various scenarios for the cracked overhung rotor system in this work. For a relatively low angular acceleration rate $\alpha = 25{\text{ rad/s}}^{2}$, it is observed that Po-BW zones remain almost the same for the entire range of unbalance force excitation angles. However, the width of some of these zones tends to narrow down at certain $\beta$ ranges compared to others. For instance, it is observed that at certain rotational frequency ($f \approx 54.5 Hz$), the Po-BW zone intensity tends to shrink at unbalance excitation angle range of $1 \le \beta \le 2.5$. This finding corresponds well to those in earlier research work^46^, where the open crack model was utilized. For a relatively high angular acceleration rate, it is observed that recurrence and width of Po-BW zones vary substantially along a different range of unbalance excitation angles. Coincidentally for both low and high angular acceleration rates, it is observed that width and recurrence of Po-BW zones tend to shrink or disappear at a relatively similar $\beta$ range $1.5 \le \beta \le 2.5$ for lower rotational speeds and $0.5 \le \beta \le 1.5$ for higher rotational speeds. Nevertheless, it is observed that for the higher angular acceleration rate, more Po-BW zones are captured. Further, it is observed that Po-BW zones, along with FW resonance speed, tend to shift to a higher frequency range for higher rotor angular acceleration rate of $\alpha = 50{\text{ rad/s}}^{2}$.

**Figure 6:**
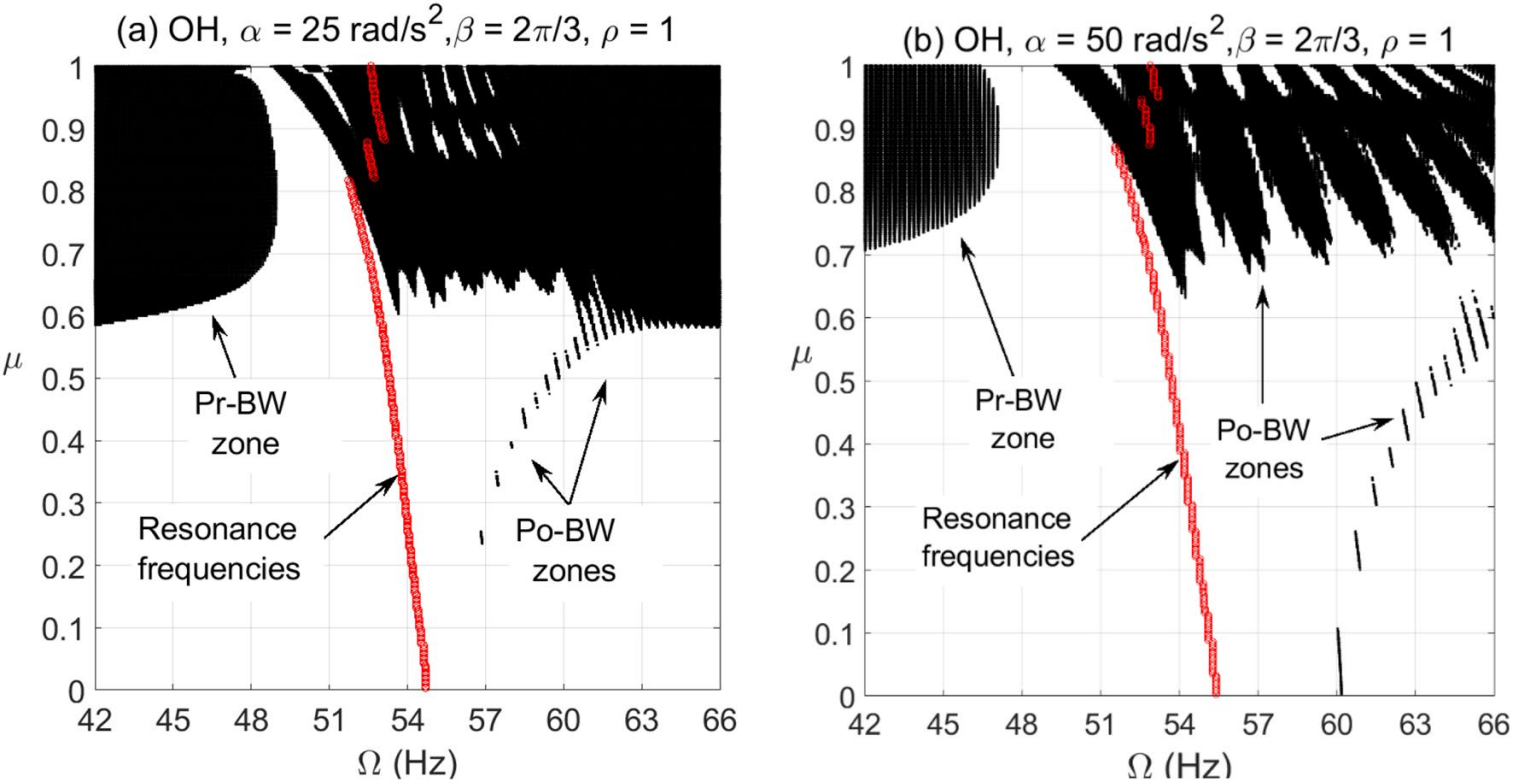
BW zones of rotational speeds at varying normalized crack depths of overhung cracked rotor system in (**a**) at $\alpha = 25{\text{ rad/s}}^{2}$ and in (**b**) at $\alpha = 50{\text{ rad/s}}^{2}$ for $\beta = {{2\pi } \mathord{\left/ {\vphantom {{2\pi } 3}} \right. \kern-\nulldelimiterspace} 3}rad$ and $\rho = 1$.

**Figure 7:**
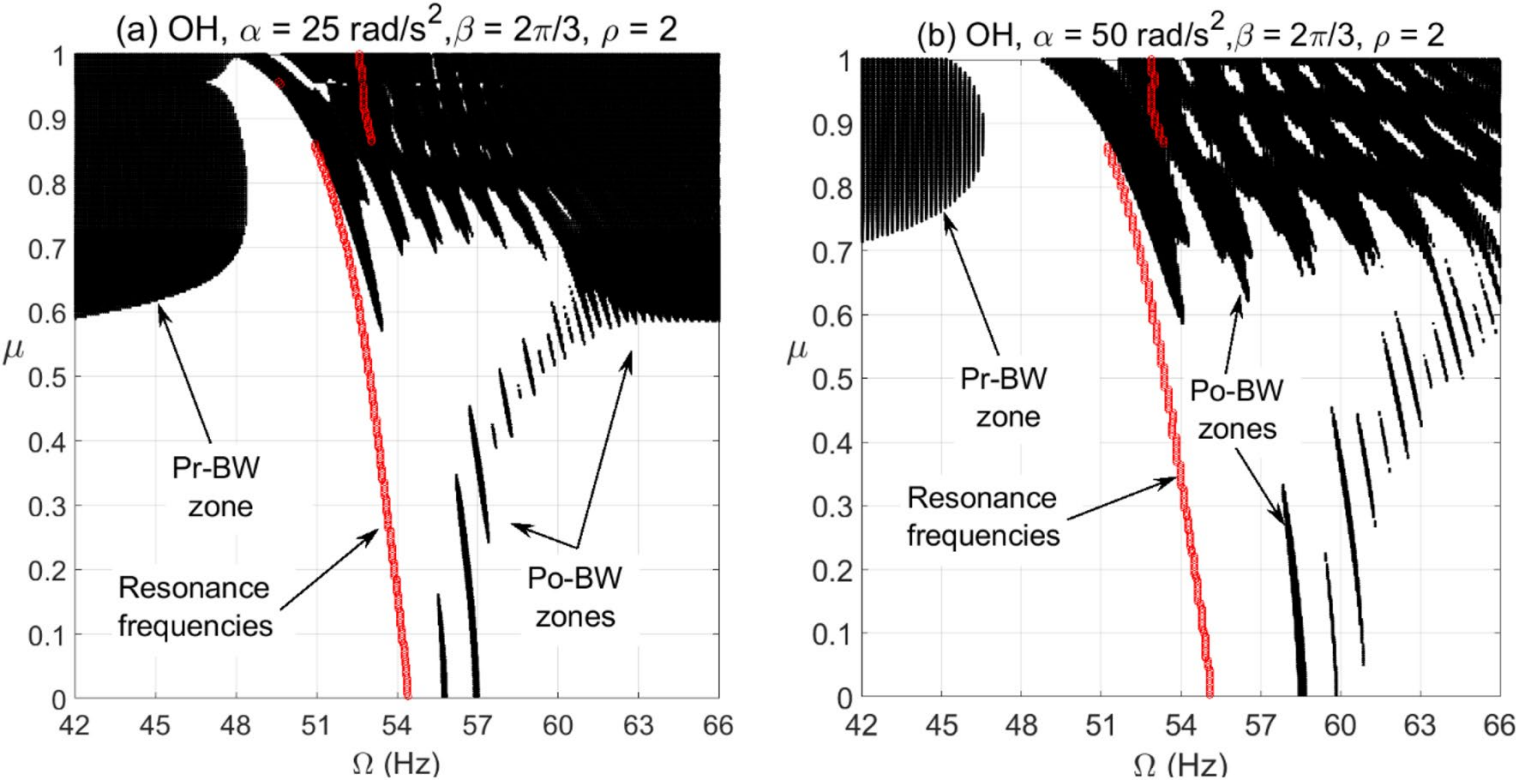
BW zones of rotational speeds at varying normalized crack depths of overhung cracked rotor system in (**a**) at $\alpha = 25{\text{ rad/s}}^{2}$ and in (**b**) at $\alpha = 50{\text{ rad/s}}^{2}$ for $\beta = {{2\pi } \mathord{\left/ {\vphantom {{2\pi } 3}} \right. \kern-\nulldelimiterspace} 3}rad$ and $\rho = 2$.

**Figure 8:**
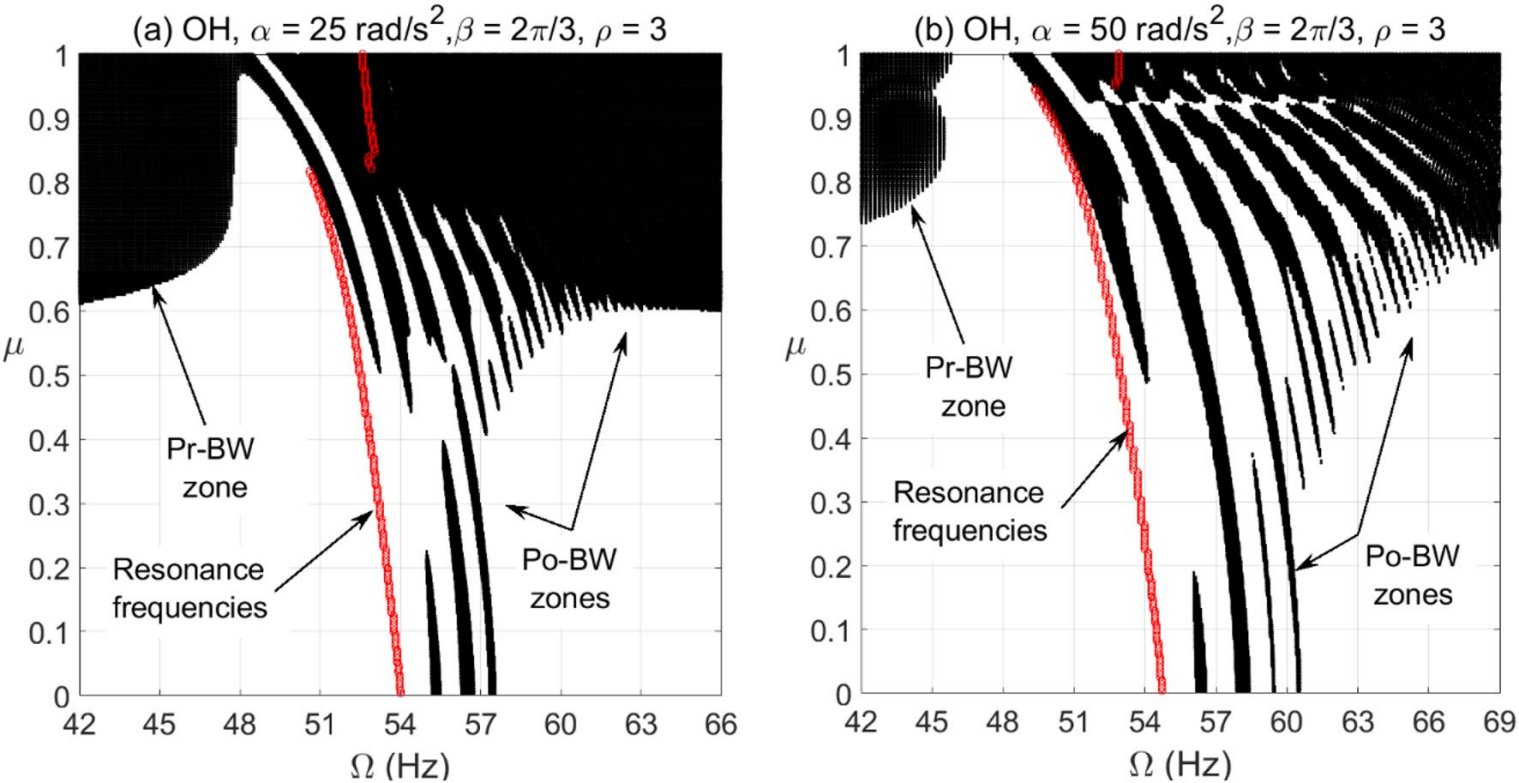
BW zones of rotational speeds at varying normalized crack depths of overhung cracked rotor system in (**a**) at $\alpha = 25{\text{ rad/s}}^{2}$ and in (**b**) at $\alpha = 50{\text{ rad/s}}^{2}$ for $\beta = {{2\pi } \mathord{\left/ {\vphantom {{2\pi } 3}} \right. \kern-\nulldelimiterspace} 3}rad$ and $\rho = 3$.

For the cracked overhung rotor, directional whirl response was evaluated against various crack depth ratios for different bearing conditions. These simulations were carried out for $\alpha = 25{\text{ rad/s}}^{2}$ and $\alpha = 50{\text{ rad/s}}^{2}$ angular acceleration rates and the results are depicted in Figs. 6, 7 and 8. These results show the directional whirl response of the cracked rotor at various crack depth ratios with isotropic bearing conditions ($\rho = 1$) in Fig. 6, $\rho = 2$ in Fig. 7, and $\rho = 3$ in Fig. 8. We notice that for both angular acceleration rate cases, for $\rho = 1$, the Po-BW zones only appear at very high crack depth ratios regardless of angular acceleration rates, see Fig. 6. However, the recurrence and intensity of Po-BW zones at a lower angular acceleration rate is relatively higher. Another interesting finding is the appearance of Pr-BW zones for both angular acceleration rates at higher crack depth ratios. The excitation zone of Pr-BW is more intensified in the case of lower acceleration rate and it takes place even at a lower crack depth ratio $\left( { \mu \approx 0.58} \right)$ when compared to higher rotor acceleration rate case ($\mu \approx 0.71)$. These observations show that the breathing crack can excite the Po-BW zones in the overhung cracked rotor at isotropic bearing condition, but mostly at relatively high crack depths.

Now, by introducing bearing anisotropy condition of $\rho = 2$ as in Fig. 7, we observe that Po-BW precession also appears in the low crack depth ratio range with more recurrent and wider zones in the case of higher angular acceleration rate $\alpha = 50{\text{ rad/s}}^{2}$. For higher crack depth ratio values, we notice that there is hardly any change in Po-BW zones when compared to the isotropic bearing condition. This is mainly attributed to the fact that Po-BW precession is mostly dominated by the effect of the large breathing crack rather than the bearing’s stiffness anisotropy at that level. In addition, we also notice the appearance of Pr-BW zones with a very similar intensity as with the isotropic bearing condition. Nevertheless, it is important to mention that such phenomena of Pr-BW were not observed in the crack-free overhung shaft. Hence, this could also be used as an indication of the presence of a crack in the system.

For the case with a higher bearing anisotropy ratio ($\rho = 3$) shown in Fig. 8, one may observe that more of the Po-BW recurrence and extension takes place with both low and high angular acceleration rates, with relatively more zones in the case of a higher angular acceleration rate. This is an interesting indication whereby variations in bearing conditions could also be monitored and trended to enable damage diagnostics and correlation. It is also observed that the appearance of Pr-BW zones for higher angular acceleration rate $\alpha = 50{\text{ rad/s}}^{2}$ shrinks if compared to previous cases shown in Figs. 6 and 7. This can be additionally employed as an additional characteristic for monitoring bearing conditions. It is to be noted however that more theoretical simulations backed up by more experimental observations will need to be further explored in order to confirm this observation.

**Figure 9:**
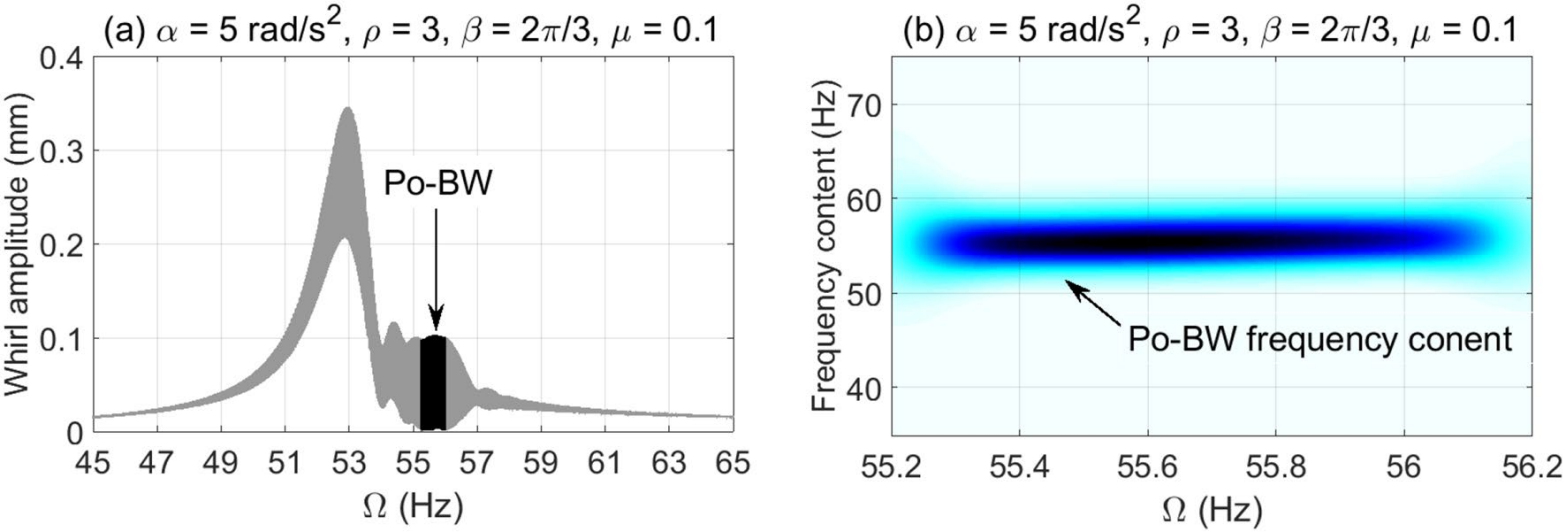
Po-BW zones of rotational speeds of an overhung cracked rotor system shown in the whirl amplitudes in (**a**), and the corresponding wavelet spectrum transform of the second Po-BW zone in (**b**) at $\mu = 0.1$, $\alpha = 5{\text{ rad/s}}^{2}$, $\beta = {{2\pi } \mathord{\left/ {\vphantom {{2\pi } 3}} \right. \kern-\nulldelimiterspace} 3}rad$, and $\rho = 3$.

**Figure 10:**
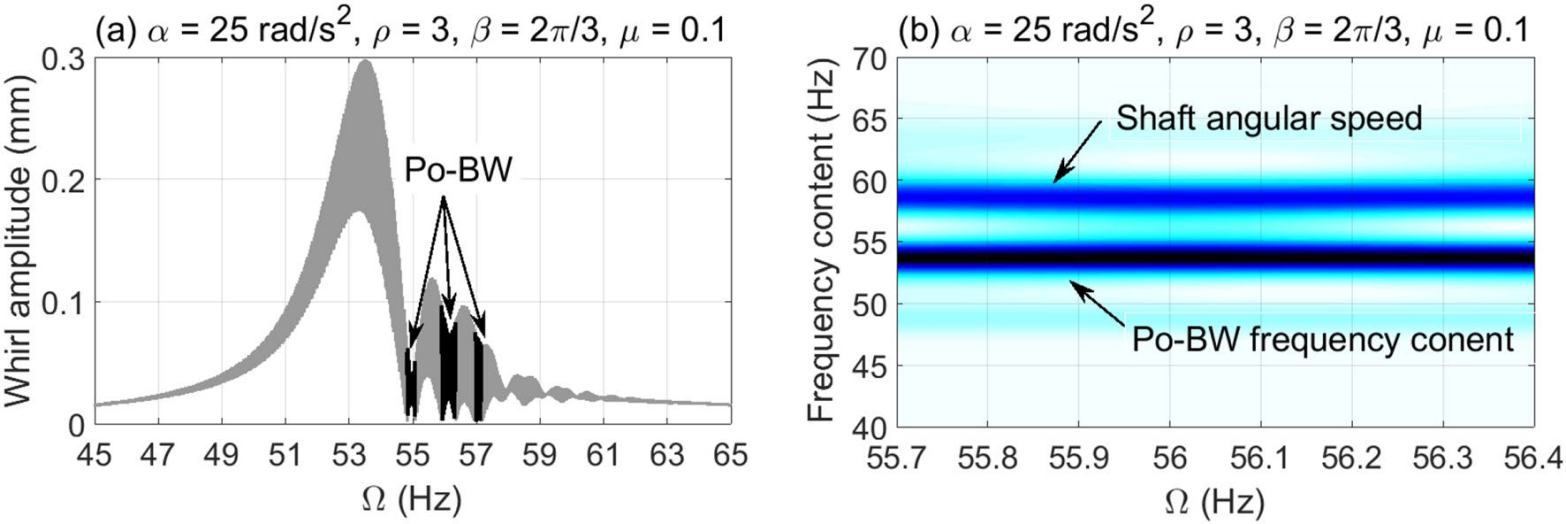
Po-BW zones of rotational speeds of an overhung cracked rotor system shown in the whirl amplitudes in (**a**), and the corresponding wavelet spectrum transform of the second Po-BW zone in (**b**) at $\mu = 0.1$, $\alpha = 25{\text{ rad/s}}^{2}$, $\beta = {{2\pi } \mathord{\left/ {\vphantom {{2\pi } 3}} \right. \kern-\nulldelimiterspace} 3}rad$, and $\rho = 3$.

**Figure 11:**
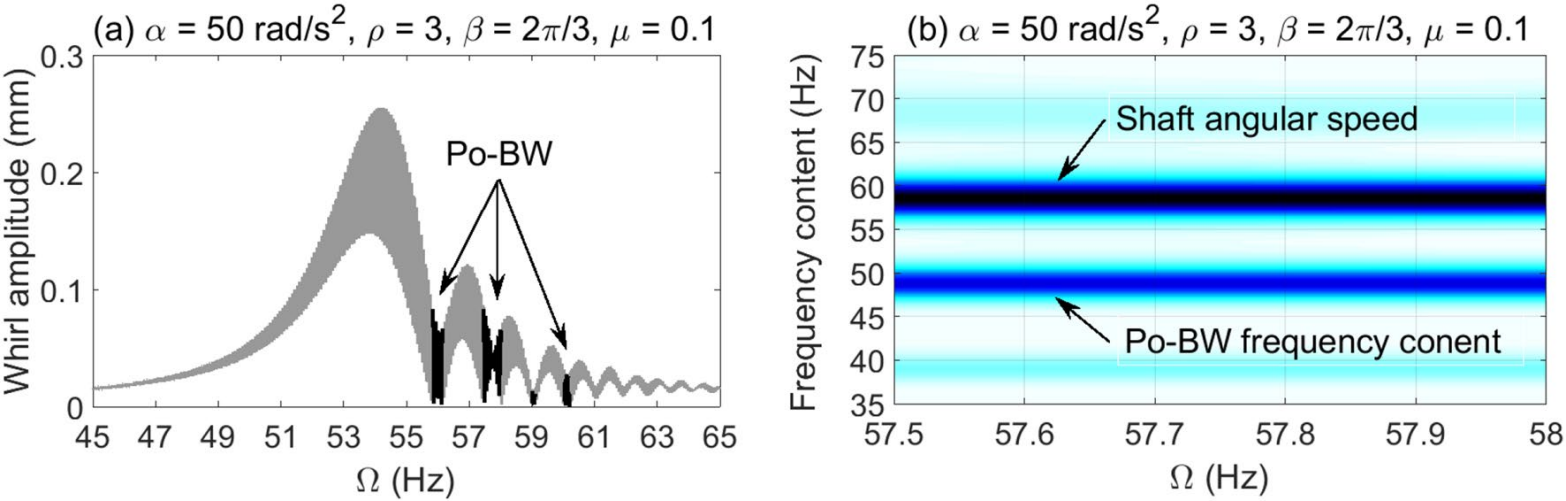
Po-BW zones of rotational speeds of an overhung cracked rotor system shown in the whirl amplitudes in (**a**), and the corresponding wavelet spectrum transform of the second Po-BW zone in (**b**) at $\mu = 0.1$, $\alpha = 50{\text{ rad/s}}^{2}$, $\beta = {{2\pi } \mathord{\left/ {\vphantom {{2\pi } 3}} \right. \kern-\nulldelimiterspace} 3}rad$, and $\rho = 3$.

The dynamic characteristics of Po-BW phenomena are further investigated using numerical whirl response and wavelet spectrum transform for overhung rotor with anisotropic bearing conditions $\rho = 3$. Accordingly, the dynamic whirl responses were computed for $\mu = 0.1$ crack depth ratio and for three different angular acceleration rates, results of which are reflected in Fig. 9, 10 and 11. For Fig. 9, the whirl response was simulated at a low angular acceleration rate of $\alpha = 5{\text{ rad/s}}^{2}$. In Fig. 9a it can be observed that Po-BW precession takes place immediately following the FW resonance curve and the minima of subsequent local transient peaks (similar findings were obtained in earlier works^45,57^ for single and double-disk systems with an open crack model) at the frequency range of $f \approx 55 - 56 Hz$. By applying the wavelet transform to the whirl response, it is interesting to note that the wavelet spectrum indicates that the frequency content of Po-BW nearly overlaps with the shaft rotational frequency as shown in Fig. 9b.

Figure 10a represents the whirl response at $\alpha = 25{\text{ rad/s}}^{2}$ for the crack depth ratio of $\mu = 0.1$, where three Po-BW zones were captured. This corresponds to the finding as reflected in Fig. 8a at a corresponding crack depth ratio of $\mu = 0.1$. After examining the wavelet spectrum of the whirl response, it is noticed that Po-BW of Fig. 10b has lower frequency content than the shaft rotational frequency. This Po-BW frequency content is close to the critical forward whirl frequency $\Omega_{fw} \cong 53.5{\text{ Hz}}$.

For the case with the higher angular acceleration rate of $\alpha = 50{\text{ rad/s}}^{2}$ in Fig. 11, simulation results are shown at similar crack depth ratio $\mu = 0.1$ to that in Fig. 9 and 10. The Po-BW zones were found matching with Fig. 8b in terms of recurrence and excitation. The wavelet spectrum corresponding to the second Po-BW zone in Fig. 11a is shown in Fig. 11b. Lower frequency contents in Po-BW zone than the shaft rotational frequency at the Po-BW is observed. This Po-BW frequency content is also lower than the critical forward whirl frequency $\Omega_{fw} \cong 53.5{\text{ Hz}}$. It is clear that increasing angular acceleration rates shift the frequency content of the Po-BW zones to lower values. At high angular acceleration rates the frequency content due to Po-BW becomes lower than the critical forward whirl frequency $\Omega_{fw} \cong 53.5{\text{ Hz}}$, even though Po-BW zones occur at higher shaft rotational frequencies.

**Figure 12:**
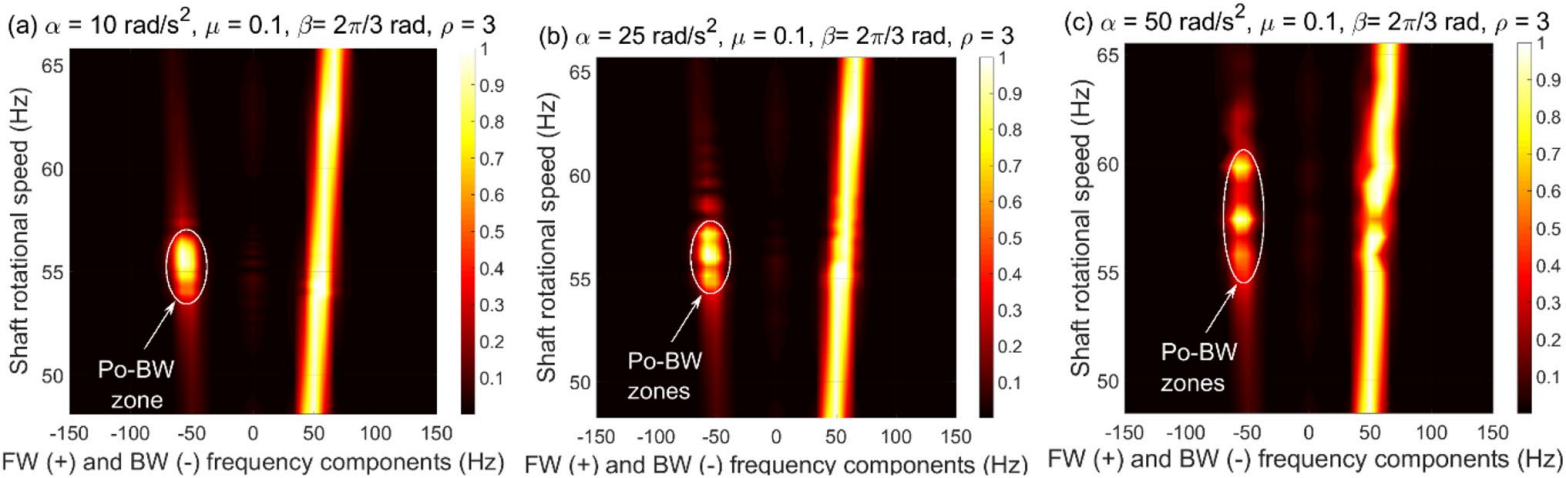
FSA plots for the Po-BW zones of rotational speeds at $\mu = 0.1$, $\beta = {{2\pi } \mathord{\left/ {\vphantom {{2\pi } 3}} \right. \kern-\nulldelimiterspace} 3}rad$ and $\rho = 3$ in (**a**) for $\alpha = 5{\text{ rad/s}}^{2}$, in (**b**) for $\alpha = 25{\text{ rad/s}}^{2}$, and in (**c**) for $\alpha = 50{\text{ rad/s}}^{2}$.

The capability of FSA is also investigated to verify the presence of these Po-BW zones for the accelerated overhung rotor at various angular acceleration rates as demonstrated in Fig. 12. It is evident from the figure that FSA is able to capture the Po-BW zones at specified rotation frequencies as indicated in the whirl response, Figs. 9a and 11a. For instance, Fig. 12b clearly demonstrates the ability of FSA in capturing Po-BW zones for the corresponding whirl response plots of Fig. 10a, which occur at rotational speed frequencies of approximately 55–57 Hz. The same is also applicable for the FSA plot in Fig. 12c and the whirl response plotted in Fig. 11a, where the frequency zone is defined in the range of 56*–*60* Hz*.

**Figure 13:**
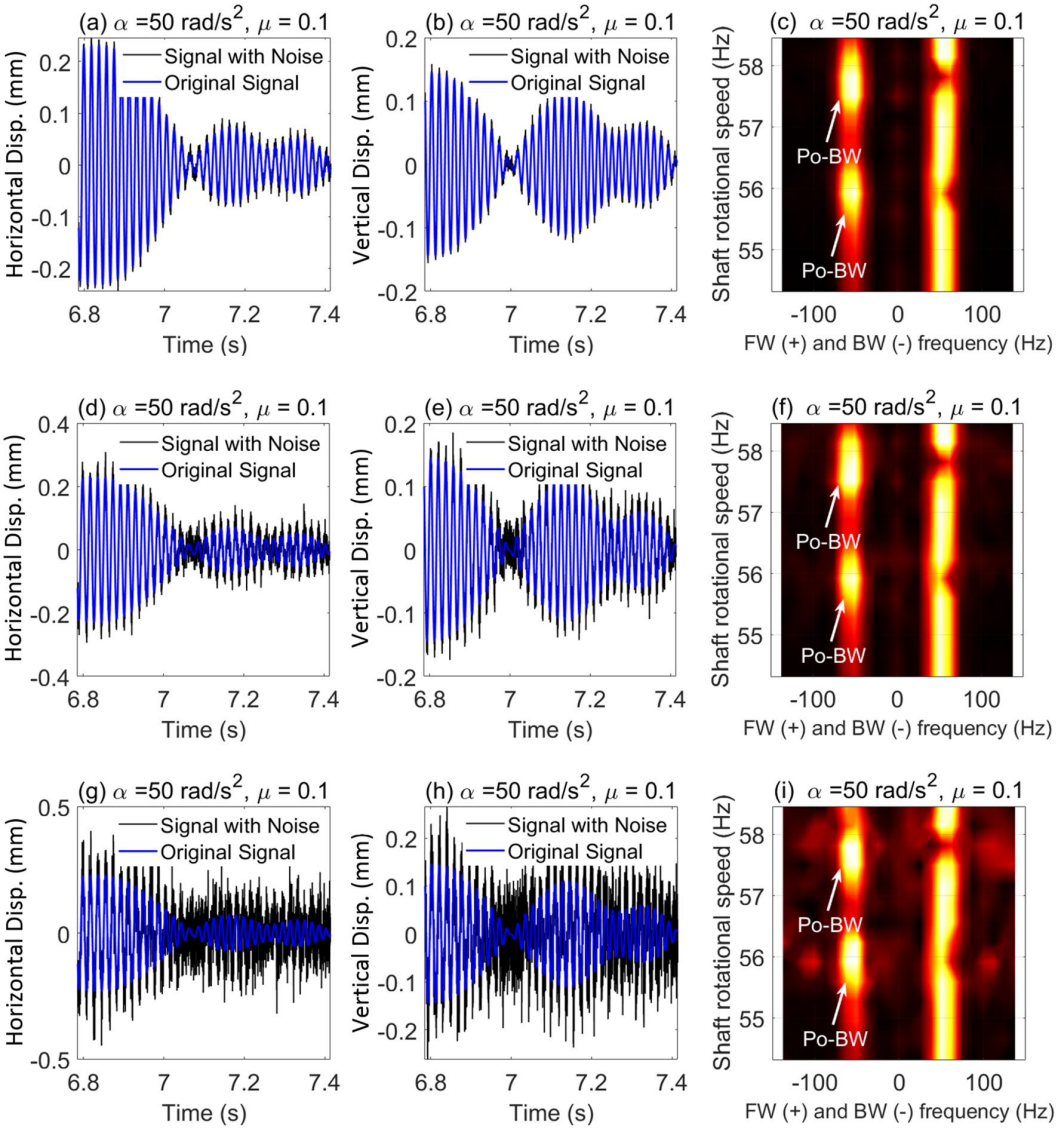
Incorporation of noise in displacement response and FSA plots for the cracked overhung rotor in element 5, at SNR = 20 dB in (**a**–**c**); SNR = 10 dB in (**d**–**f**); and SNR = 1 dB in (**g**–**i**).

In order to better replicate the conditions typically encountered in real rotor system applications, it is necessary to consider data contamination with noise. Sensors such as proximity probes and accelerometers can be affected by noise from operating environments, which exceed the noise level typically seen in specialized research labs. Here, we added artificial random noise to the simulation data for the case that was previously shown Fig. 11 in order to achieve various Signal-to-Noise Ratio (SNR) values. For the relatively high SNR value (SNR = 20 dB), it is evident from Fig. 13a–c that the impact is almost negligible and that Po-BW zones are clearly captured and observed at the corresponding angular frequencies in Fig. 11. By further reducing the SNR to 10 dB (in Fig. 13d–f, the impact can still be seen as negligible. And, by considering an extreme scenario of SNR = 1 dB in Fig. 13g–i, it is interesting to note that the Po-BW zone can be still robustly captured using the FSA analysis. This once again proves the robustness of FSA as a powerful tool in capturing these new types of BW zones in transient rotor operations.

**Figure 14:**
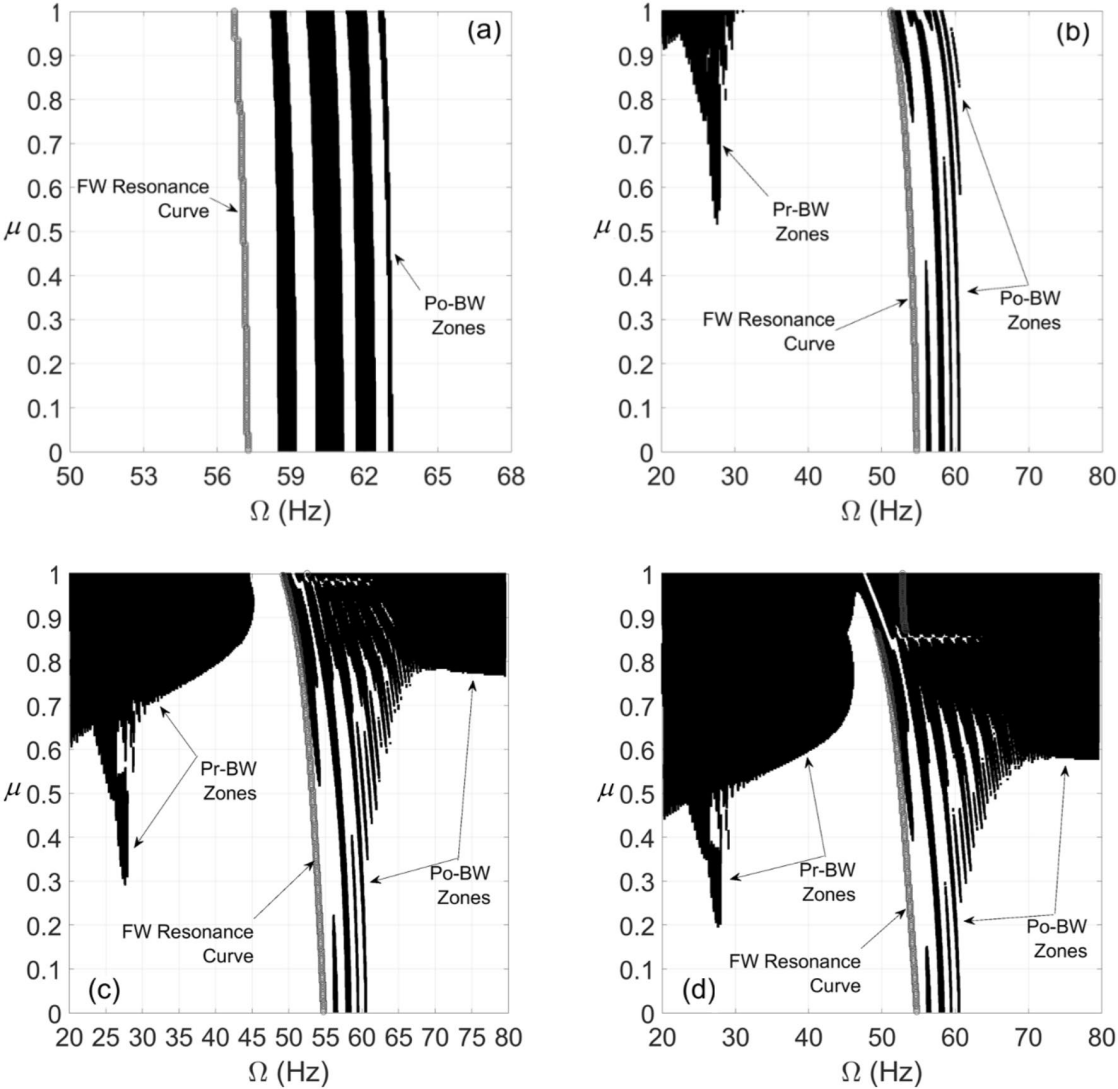
BW zones of rotational speeds at varying normalized crack depths of an overhung cracked rotor system for the crack located (**a**) at the first element, (**b**) at the second element, (**c**) at the third element, and (**d**) at the fourth element for $\alpha = 50{\text{ rad/s}}^{2}$, $\beta = {{2\pi } \mathord{\left/ {\vphantom {{2\pi } 3}} \right. \kern-\nulldelimiterspace} 3}rad$ and $\rho = 3$.

In all previous results, directional whirl response has been assessed for the crack located at element 5 of the FE model. However, the location of the crack along the span of the overhung rotor could have a significant impact on Po-BW signature. Therefore, this study assesses the effect of the crack location on Pr-BW and Po-BW behavior, the results of which are shown in Fig. 14. The figure shows whirl response plots for the cracked overhung rotor with the crack located at the first, second, third, and fourth elements one at a time for FE simulation. It is observed that Po-BW zones appear wider for the crack located in the first element compared to other locations. It is also interesting to note that Pr-BW zone is not evident in the results when the crack is in the first element. By evaluating Fig. 14b with the crack located in the second element, it can be observed that Po-BW zones are narrower compared to Fig. 14a and that Pr-BW zones start to appear at relatively high values of the crack depth ratio. Nevertheless, the intensity of Pr-BW zones is much smaller compared with Pr-BW zones for the cases with cracks located in the third and fourth elements. For subsequent Fig. 14c,d with cracks located at the third and fourth elements, respectively, we notice a gradual increase in Pr-BW and Po-BW intensities by further shifting the crack location closer to the rotor midspan and at relatively high values of the crack depth ratios. The Po-BW recurrence and intensities remain roughly the same at the low crack depth ratio range. Generalizing the above findings, it can be said that the behavior of both Pr-BW and Po-BW zones will need to be assessed together in order to diagnose the location of crack. For high intensity Po-BW zones, the crack is more likely to be located closer to the left bearing. As the crack position is shifted toward the center, Pr-BW zone undergoes significant changes, and hence it could serve as a discriminating feature for determining the possible location of the crack. Further research is needed to achieve accurate correlations between crack locations and backward precession zones.

**Figure 15:**
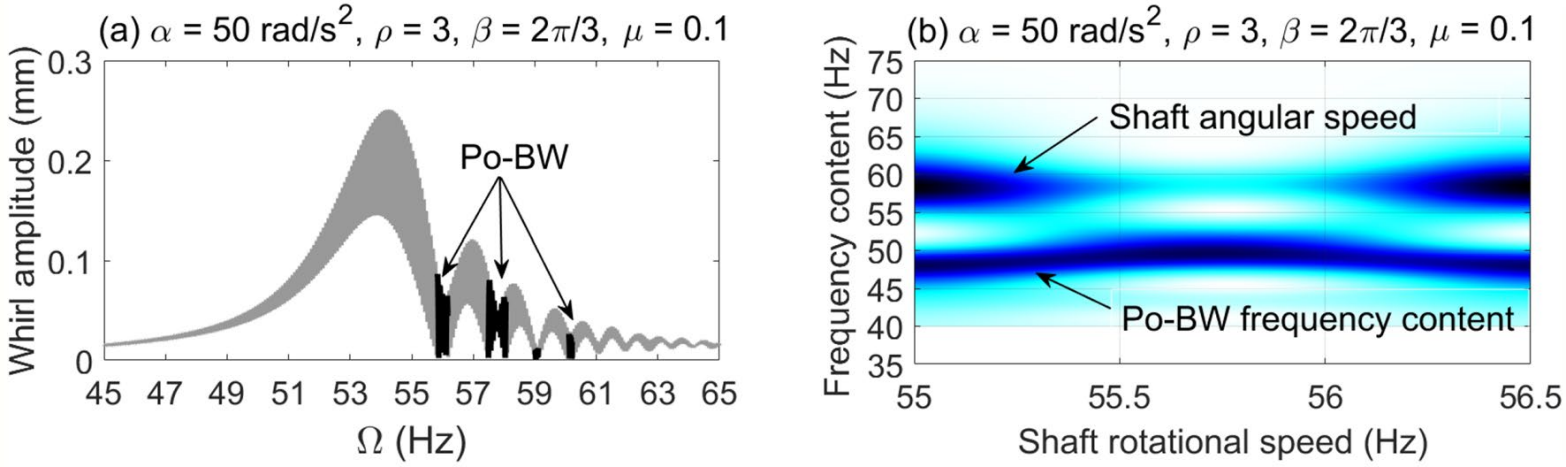
Po-BW zones of rotational speeds of a cracked overhung rotor at element 3 shown in the whirl amplitudes in (**a**), and the corresponding wavelet spectrum transform of the second Po-BW zone in (**b**) at $\mu = 0.1$, $\alpha = 50{\text{ rad/s}}^{2}$, $\beta = {{2\pi } \mathord{\left/ {\vphantom {{2\pi } 3}} \right. \kern-\nulldelimiterspace} 3}rad$, and $\rho = 3$.

In the remaining analysis the crack location is assumed to be in element 3 between the bearings. For this crack location the Po-BW zones in the whirl response are shown in Fig. 14c. Therefore, the corresponding whirl response and wavelet transform spectrum of the frequency content at $\mu = 0.1$ are shown in Fig. 15. Four Po-BW zones get excited as shown in Fig. 15a with frequency content on the left and the wavelet frequency spectrum on the right in Fig. 15b. It is observed that the frequency content in this Po-BW zones is less than the resonance frequency of the system.

**Figure 16:**
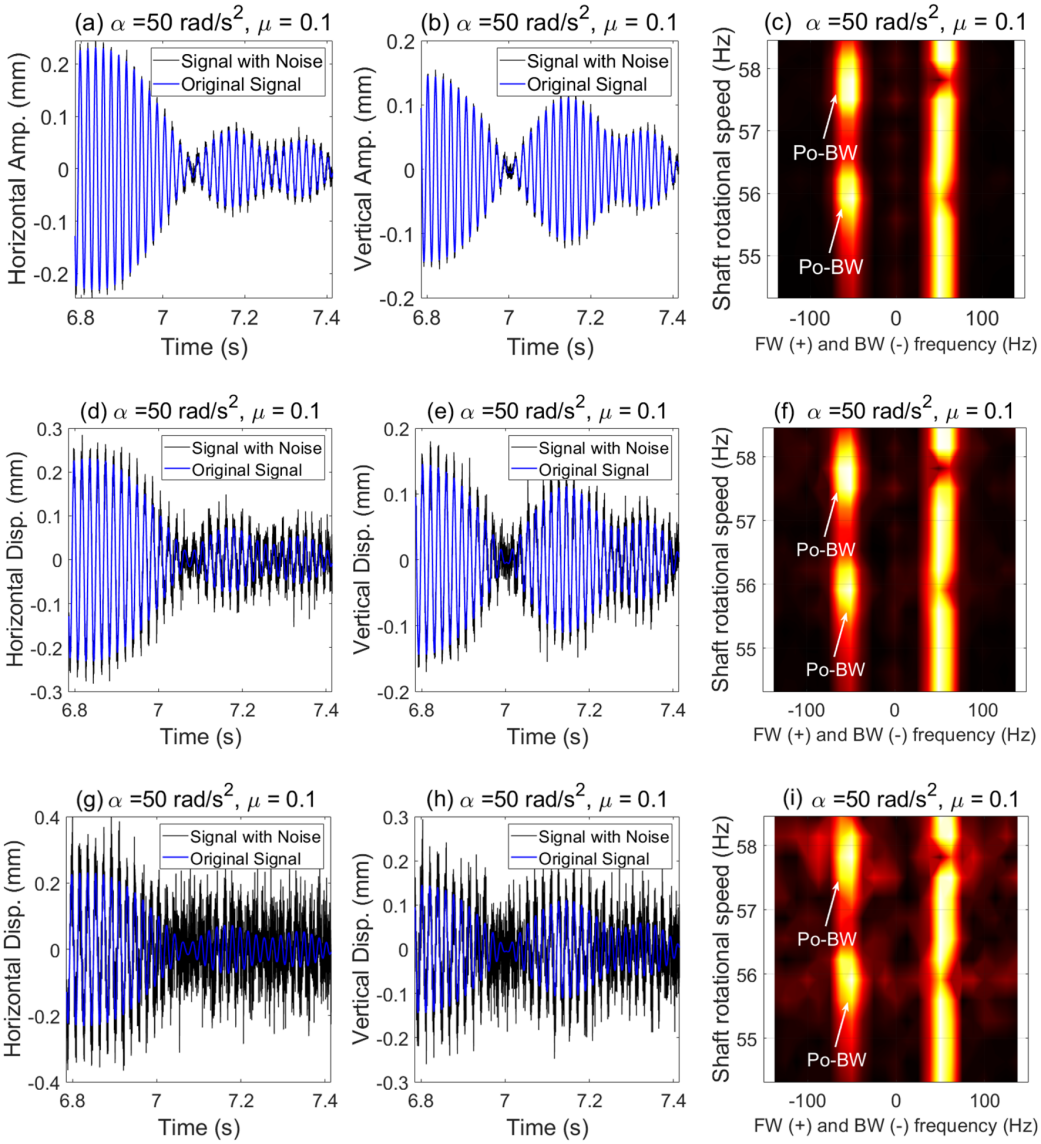
Incorporation of noise in displacement response and FSA plots for the cracked overhung rotor in element 3, for SNR = 20 dB in (**a**–**c**); SNR = 10 dB in (**d**–**f**); and SNR = 1 dB in (**g**–**i**).

Similar to the results in Fig. 13 where the crack location was in element 5, for crack location in element 3, an artificial random noise is added here to the simulation response data for the case in Fig. 15. For high SNR value (SNR = 20 dB) in Fig. 16a–c, the influence of the added noise is found to be negligible on the Po-BW zones which are still clearly observed as shown in the FSA plot. In Fig. 16d–f, the effect of reducing the SNR to 10 dB is also found to be negligible in capturing the Po-BW zones by the FSA analysis. For SNR = 1 dB which is indeed a very high level of noise induced in the response (as shown in Fig. 16g–i), the Po-BW zone are still clearly captured by the FSA analysis. This once again demonstrates the robustness of FSA as a powerful tool in capturing the Po-BW zones in the whirl response.

## Conclusion

Overhung rotor systems constitute a wide variety of heavy-duty industrial applications. Therefore, the post-resonance backward whirl (Po-BW) phenomenon, which has been observed in geometrically symmetrical rotor systems is further investigated here with overhung rotor systems. The contribution of this study is to confirm that the existence of Po-BW is not only observed with rotors that are geometrically symmetric about the shaft’s mid-span but also with geometrically unsymmetrical configurations like overhung rotors. The study is based on numerical simulation of the linear-time-varying FE model equations of motion of accelerated intact and cracked overhung rotor systems considering isotropic and anisotropic bearings conditions.

For the intact version of the considered overhung rotor system with anisotropic bearings, the existence of the Po-BW precession is confirmed here and found to be also affected by the gyroscopic effect, unbalance force vector orientation and acceleration rate. However, the Po-BW phenomena is not observed to be affected by the gravity force. For the cracked overhung rotor system with a breathing crack, the excitation of Po-BW precession was also confirmed, where it is found to be affected by the unbalance force vector angles and the acceleration rates. The impact of the bearings’ condition on the obtained Po-BW zones of the cracked overhung rotor system has been also studied, and it is observed that stiffness anisotropy results in significant excitation of Po-BW zones, especially at lower crack depths.

The wavelet transform spectrum analysis of the whirl response in the Po-BW zones shows a significant effect of the acceleration rate on the frequency content. Moreover, employing FSA has also confirmed the excitation of Po-BW zones in all the aforementioned cases. Furthermore, the crack location along the rotor axis was found to be profoundly affecting the Po-BW excitation, intensity and recurrence.

The important finding here is that the Po-BW zones in overhung rotor system are highly sensitive to bearing anisotropy and crack propagation compared with the Pr-BW zones. Therefore, the probability of exciting Po-BW zones by the crack and bearing anisotropy is much higher than that with Pr-BW zones. This observation points to the exciting prospect of being able to establish a robust Po-BW based damage detection method for overhead rotor systems. Further research is being pursued along these lines.

## Supplementary Information

Supplementary Information.
