# Supplementary material for: Post-resonance backward whirl analysis in cracked overhung rotors
Source: Sci Rep. 2022 May 20;12:8517. doi: 10.1038/s41598-022-12068-w (PMC9123228; doi:10.1038/s41598-022-12068-w)
Supplement: Supplementary file 1 — Supplementary Information. [file 41598_2022_12068_MOESM1_ESM.docx]

**Supplementary Note**

**List of Abbreviations and Symbols**

| *BW* | Backward whirl |
| --- | --- |
| DOF | Degree of freedom |
| EOM | Equations of motion |
| FE | Finite element |
| FSA | Full-spectrum analysis |
| *FW* | Forward whirl |
| HB | Harmonic balance |
| LFM | Linear fracture mechanics |
| LTV | Linear time-variant |
| OH | Overhung rotor |
| Pr-BW | Pre-resonance backward whirl |
| Po-BW | Post-resonance backward whirl |
| SIF | Stress intensity factors |
| VCP | Vector cross product |
| $\alpha$ | Angular acceleration |
| *β* | Angle of the unbalance force vector with respect to the crack |
| *α_ω_, β_ω_* | Phase angles of the forward and backward rotating vectors at frequency ω |
| *δ* | Mass proportional (external) viscous damping coefficient |
| *η* | Stiffness proportional (internal) viscous damping coefficient |
| *μ* | Normalized crack depth with respect to the radius of the shaft |
| $\rho=k_{yy}/k_{xx}$ | Ratio representing bearing stiffness anisotropy |
| *ρ_s_* | Density of the shaft material |
| $\theta(t)$ | Angular rotation |
| $\Omega(t)$ | Angular rotation velocity |
| *ω* | Angular frequency |
| **C**_R_ | Internal material damping matrix |
| $F_{u}(t)$ | Unbalance force vector |
| $F_{g}$ | Gravity force vector |
| **G** | Global gyroscopic matrix |
| **k**(t) | $8\times8$stiffness matrix of the cracked element  |
| $K_{S}$ | Global stiffness matrix of the shaft in the rotating coordinates |
| $K_{C}$ | Skew-symmetric circulation matrix |
| $K_{G}$ | Skew-symmetric stiffness matrix contribution |
| $K_{T}$ | global stiffness matrix |
| **M** | Global mass matrix |
|  | Acceleration vector |
|  | Velocity vector |
| $q(t)$ | Displacement vector |
| **r** | Radial position vector of the shaft centerline due to deflections |
| $c_{xx}$, $c_{yy}$ | Bearing damping coefficients in horizontal and vertical directions |
| *E* | Elastic modulus of the rotor material |
| *e* | Unbalance mass eccentricity |
| *f_i_* | Elemental unbalance force vector |
| *h* | Radial crack depth |
| $I_{\bar{X}}(t)$ | Moment of inertia about fixed centroidal $\bar{X}$-axis  |
| $I_{\bar{Y}}(t)$ | Moment of inertia about fixed centroidal $\bar{Y}$-axis  |
| $I_{\bar{X}\bar{Y}}$*(t)*  | Product of inertia |
| $k_{yy}$, $k_{xx}$ | Bearing stiffness in vertical and horizontal directions |
| *L* | Shaft length |
| *l* | Element length |
| $m$ | Unbalance mass |
| *R* | Shaft radius |
| $R_{\omega+}$*,* $R_{\omega-}$ | Magnitudes of forward and backward rotating vectors at frequency *ω* |
| $t$ | Time, s |
| $u$ | Displacement along the stationary *X*-axis |
| $v$ | Displacement along the stationary *Y*-axis |
| $x,y$ | Rotating coordinate axes |
| $X,Y$ | Fixed coordinate axes |
| $\bar{X},\bar{Y}$ | Stationary centroidal coordinate axes |
| $\bar{x},\bar{y}$ | Rotating centroidal coordinate axes |
| *z* | Resultant whirl amplitude |
